# An Improved YOLOv5-Based Lightweight Submarine Target Detection Algorithm

**DOI:** 10.3390/s23249699

**Published:** 2023-12-08

**Authors:** Likun Mei, Zhili Chen

**Affiliations:** School of Optoelectronic Engineering, Xi’an Technological University, Xi’an 710021, China; yl04231214@163.com

**Keywords:** C3_DS, SA-net, light weight

## Abstract

Submarine recognition plays a critical role in maritime security and military defense. However, traditional submarine recognition algorithms face limitations in feature representation capability and robustness. Additionally, deploying deep learning methods on embedded and mobile platforms presents a bottleneck. To address these challenges, we propose an innovative and practical approach—an improved YOLOv5-based lightweight submarine automatic recognition detection algorithm. Our method leverages the Feature Pyramid based on MobileNetV3 and the C3_DS module to reduce computation and parameter complexity while ensuring high precision in submarine recognition. The integration of the adaptive neck from the SA-net strategy further mitigates missed detections, significantly enhancing the accuracy of submarine target detection and recognition. We evaluated our improved model on a submarine dataset, and the results demonstrate remarkable advancements in Precision, Recall, and mAP0.5, with respective increases of 8.54%, 6.02%, and 3.36%. Moreover, we achieved a notable reduction of 34.1% in parameter quantity and 67.9% in computational complexity, showcasing its lightweight effects. Overall, our proposed method introduces novel improvements to submarine recognition, addressing existing limitations and offering practical benefits for real-world deployment on embedded and mobile platforms. The enhanced performance in precision and recall metrics, coupled with reduced computational requirements, emphasizes the significance of our approach in enhancing maritime security and military applications.

## 1. Introduction

Recognizing submarines [1,2] holds significant importance within the realms of maritime security and national defense. It plays a crucial role in ensuring the safety of maritime traffic and upholding national security [3,4,5]. Traditional approaches to submarine recognition [6,7,8] heavily depend on manually crafted features and intricate algorithmic processes, leading to limitations in accuracy and efficiency, especially in large-scale and complex scenarios. The imperative is to achieve highly automated submarine target recognition, enhancing both accuracy and real-time performance while concurrently minimizing computational costs and memory overhead [9].

Some scholars have conducted research on traditional submarine recognition methods. Xu Yinghao [10] and others conducted simulation studies on the sea surface wave and target characteristics of the hydrodynamic wake of a fully appended submarine by combining laser radar imaging and pattern recognition technology. Munteanu [11] and others have successfully achieved high-precision detection of floating and underwater sea mines, offering potential application possibilities in real-time scenarios, especially in regions with frequent economic activities along the Black Sea coastline. Yi Zhihang [12] and others achieved multimodal recognition in submarine recognition by using different sensors and detection methods at different stages of submarine motion. Manjula R B [13] and their team proposed a sensor deployment design scheme based on Particle Swarm Optimization, aiming for optimal coverage with the minimum number of sensor nodes for anti-submarine detection in Underwater Acoustic Sensor Networks. The scheme was simulated and analyzed to study the impact of various factors on the coverage area. Zhu L [14] and colleagues conducted an in-depth literature review on the evolution of mainstream object detection algorithms, proposing an approach based on modifying the YOLOv5 model by altering receptive fields through the addition of asymmetrical pooling layers. This modification enhances accuracy while maintaining speed. The comprehensive evaluation of its performance across various parameters and scenarios is presented, offering insights into future research directions. Yadav, P.K [15] and colleagues present the application of YOLOv5m and a customized Unmanned Aircraft System (UAS) for detecting and locating volunteer cotton (VC) plants in the middle of cornfields. This approach achieves high accuracy and speed, demonstrating effective spot-spray applications for efficient management of boll weevil pest infestations. The research holds significant importance for assessing cotton yield and crop management, showcasing the potential application of YOLOv5 in the agricultural domain.

These studies have made certain contributions in the field of submarine recognition. However, there are issues with traditional detection algorithms, such as difficulties in feature design, poor robustness, limited generalization ability, as well as the large computational and parameter requirements of existing deep learning-based methods, and the difficulty of deploying them on embedded devices and mobile platforms [16,17]. Therefore, this paper aims to propose a lightweight submarine target recognition method based on improved YOLOv5. By optimizing the network structure and designing more compact and efficient convolution operators, we reduce the model’s parameter and computational requirements while maintaining high accuracy, in order to adapt to resource-constrained environments on embedded devices and mobile platforms, and achieve efficient and real-time submarine recognition. The improved YOLOv5-based lightweight submarine target recognition algorithm has significant advantages over traditional algorithms in terms of submarine recognition performance. Firstly, we address the issue of large parameter and computational requirements when using YOLOv5 by optimizing the network structure and parameter count, thereby improving the algorithm’s real-time performance. Secondly, we adaptively adjust the network’s weights based on the characteristics of submarine targets, allowing the network to focus more on the important features of submarine target regions, further enhancing accuracy. In summary, our algorithm not only demonstrates advantages in accuracy and real-time performance but also provides an efficient, accurate, and practical solution for submarine target recognition through improvements in lightweight design and attention mechanisms. This is of great significance to the fields of maritime security and national defense, and it provides strong technical support for the research and practical applications of submarine target recognition.

## 2. Principle of YOLOv5 Algorithm

**Figure 1:**
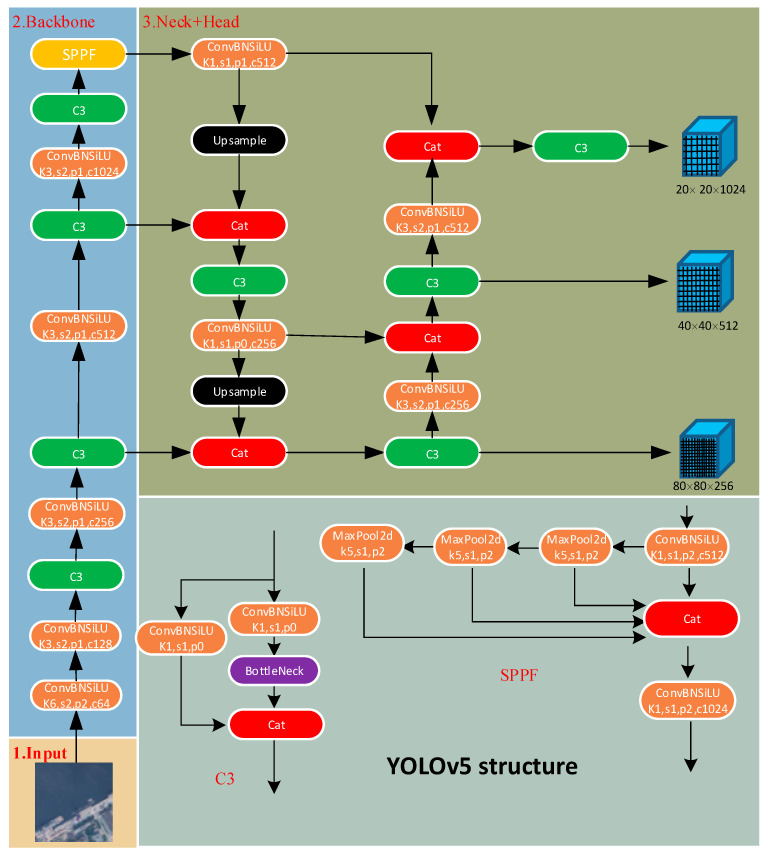
Overall architecture of YOLOv5.

YOLOv5 [18] is a popular object detection algorithm that is improved and optimized based on YOLOv4 [19]. The principle of YOLOv5 is based on the idea called “You Only Look Once” (YOLO), which treats object detection as a regression problem and predicts bounding boxes and class labels simultaneously in a single neural network, as shown in Figure 1. Compared to traditional two-stage detectors like Faster R-CNN [20], YOLOv5 adopts a simpler and more direct approach, striking a better balance between speed and accuracy. YOLOv5 introduces new data augmentation strategies such as Auto Augment and Multi-Scale Training. These strategies increase the diversity of training data, improving the model’s robustness and generalization capability. YOLOv5 adapts to different application scenarios and hardware requirements by introducing model variants of different scales, such as YOLOv5s, YOLOv5m, YOLOv5l, and YOLOv5x. This scaling approach allows users to flexibly choose models according to their specific needs, balancing speed and accuracy.

The backbone network is one of the core components of the object detection algorithm, employing a deep convolutional neural network structure and playing a vital role in extracting feature representations from input images. The backbone network gradually reduces the size of the feature maps and extracts multi-scale feature information at different levels through a series of convolutional and pooling layers. The backbone network typically consists of multiple convolutional and pooling layers. The convolutional layers slide convolutional kernels over the feature maps to extract low-level features such as edges and textures. As the depth of the network increases, the convolutional layers enhance the abstract representation capability of the input images. Pooling layers are used to reduce the size of the feature maps while preserving important feature information and reducing computational complexity. By stacking and combining convolutional and pooling layers progressively, the backbone network constructs a multi-level feature representation that facilitates capturing object information in the images effectively.

“Neck” is an intermediate layer structure that connects the backbone network and the detection head in object detection. It plays a crucial role in further processing the features extracted by the backbone network to generate feature representations suitable for object detection. Firstly, the neck implements feature fusion to integrate features from different levels of the backbone network. This fusion effectively combines feature information at different scales, enhancing the perception of multi-scale objects by the object detection algorithm. Secondly, the neck is responsible for building a feature pyramid [21] to handle objects of different scales. By introducing multiple branches or multi-level feature extraction, the feature pyramid can extract features at different levels, aiding in improving the algorithm’s robustness to scale variations and enabling effective detection and recognition of objects of different sizes. Additionally, the neck performs upsampling and downsampling operations to adjust the size of the feature maps. Upsampling can enlarge low-resolution feature maps to recover detailed information of the targets, while downsampling can reduce the size of high-resolution feature maps, increasing the receptive field and capturing a broader context.

The “head” typically refers to the last part of the network, which is responsible for processing and interpreting the features passed from the backbone network and the intermediate layer (neck) to achieve object localization and classification. The head network usually consists of a classifier, a regressor, a prior box generator, and non-maximum suppression (NMS) [22]. The classifier is the core component of the head network, which receives the features transmitted from the intermediate layer and utilizes operations such as fully connected layers, convolutional layers, and pooling layers to classify the objects within the detection boxes and output the probability distribution of object categories. The regressor is used to predict the position and size information of the detection boxes. It generates accurate regression parameters through fully connected layers or convolutional layers combined with appropriate activation functions and normalization operations. The prior box generator is responsible for generating a set of candidate detection boxes in the image based on the scale and aspect ratios of the input features, providing a reference for subsequent classification and regression. Finally, the NMS algorithm selects the best boxes among multiple overlapping detection boxes, eliminating redundancy and improving detection accuracy and recall. The goal of the head network design is to effectively convert the features extracted by the backbone network and intermediate layer into the final object detection results. Through the collaboration of the classifier, regressor, and prior box generator, the head network can accurately predict the object categories and positions. Meanwhile, the application of the NMS algorithm removes overlapping detection boxes to ensure accurate and reliable final detection results.

In summary, YOLOv5 is an efficient and accurate object detection algorithm characterized by its lightweight design, multi-scale inference, improved backbone network, and optimized data augmentation and training strategies. It exhibits significant improvements in both speed and performance, enabling fast object detection while maintaining high accuracy. YOLOv5 has broad application prospects in various real-time object detection tasks.

## 3. Improved YOLOv5

### 3.1. Feature Pyramid Based on MobileNetV3

MobileNetV3 [23] follows the design principles of reduced width, increased input resolution, and stronger non-linearity. By reducing the width of the network, which refers to reducing the number of channels in each convolution operation, the computational complexity can be decreased. Additionally, increasing the input resolution improves the model’s ability to perceive details and small objects. MobileNetV3 also introduces stronger non-linear activation functions such as Hard-Swish [24] and linear bottlenecks [25] to enhance feature representation. RELU6 is an extension of the ReLU function that limits the output to zero or above for negative input values and restricts the output to six or below for positive input values, resulting in an output range of [0, 6] as shown in Equation (1).

(1) $$ReLU6\left(x\right)=min\left(max\left(0,\;x\right),6\right)$$

This makes ReLU6 more suitable for certain application scenarios, such as object detection, where it can be used to limit the coordinate range of bounding boxes, ensuring that the bounding box positions remain within a reasonable range. In comparison to ReLU6, the *SWISH* function can provide smoother activation characteristics in some cases, which may help reduce the gradient vanishing problem during training and potentially improve model performance, as shown in Equation (2).

(2) $$SWISH\left(x\right)=x\times sigmoid\left(x\right)$$

However, SWISH function has a high computational complexity. It involves the calculation of the sigmoid function, which is relatively complex and involves exponential operations. This leads to a higher computational burden for SWISH, especially in large-scale neural networks, increasing the computational load.

(3) $$sigmoid\left(x\right)=1/\left(1+exp\left(-x\right)\right)$$

**Figure 2:**
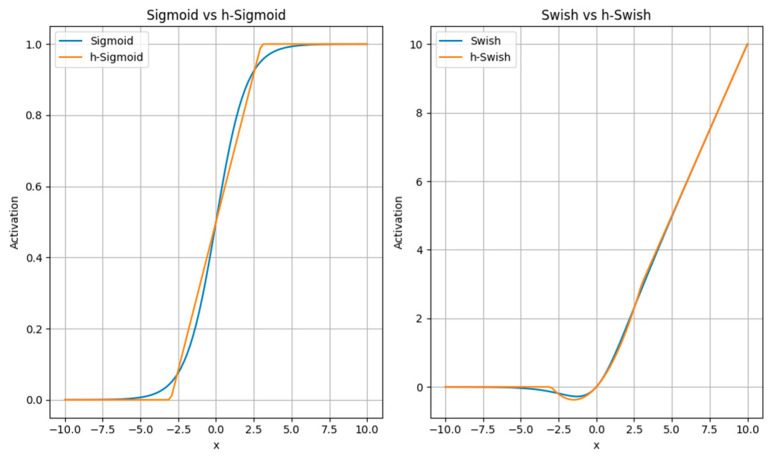
Activation function.

The “h_sigmoid” activation function is obtained by applying the ReLU6 function to the input value × + 3 and then dividing the result by 6. It has a similar shape to the sigmoid function, as shown in Figure 2 but with simpler calculations and derivative computations.

(4) $$h\_sigmoid\left(x\right)=relu6\left(x+3\right)/6$$

Replacing the sigmoid function in the swish activation function with the h_sigmoid function not only results in a similar shape to the swish function, as shown in Figure 2, but also improves inference speed and facilitates the quantization process.

(5) $$h\_swish\left(x\right)=x\times h\_sigmoid\left(x\right)$$

In terms of network architecture, MobileNetV3 utilizes grouped convolution [26] and depth-wise separable convolution [27]. Grouped convolution divides the input feature map into multiple groups and performs convolution operations on each group, then concatenates the results. By employing grouped convolution, the parameter and computational complexity can be effectively reduced. This is particularly useful when using larger convolutional neural network models with limited computational resources, such as real-time image processing on mobile devices.

Furthermore, grouped convolution can also improve the parallelism of the model to some extent, allowing for parallel computation on multiple GPUs or processors, thereby accelerating the speed of training and inference. Depth-wise separable convolution decomposes the standard convolution into two steps: depth-wise convolution and point-wise convolution, further reducing the computational complexity.

**Figure 3:**
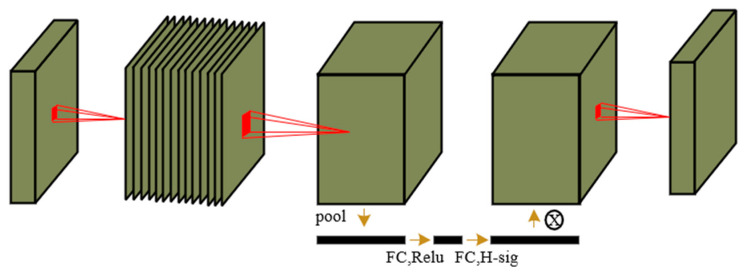
Reverse residual.

The core idea of the inverted residuals structure is to map low-dimensional feature maps to a high-dimensional space and then compress them back to a low dimension through linear projection, as shown in Figure 3. Its design goal is to address the issue of excessive parameters and computational complexity in traditional residual structures (Residuals) [28] in lightweight networks. It consists of an expansion layer and a linear bottleneck layer.

The expansion layer is used to increase the number of channels, while the linear bottleneck layer is used to reduce the dimensionality and introduce non-linear activation functions. This structure enables more effective utilization of the model’s parameters and enhances the representation capability of features, as shown in the figure. Through the inverted residuals structure, the network can perform feature extraction and non-linear transformations at a lower dimension, and then increase the feature representation capability through high-dimensional mapping and linear projection. This design reduces the number of parameters and computational complexity of the network while improving its representation capability, making lightweight networks more efficient while maintaining high performance.

**Figure 4:**
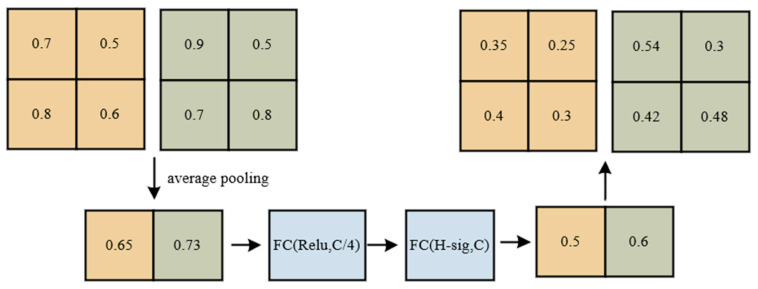
SE-net.

In addition, the Squeeze-and-Excitation (SE) [29] module is used for channel-wise attention weighting of feature maps. The SE module learns the importance weights of each channel in the feature map through global average pooling and two fully connected layers, and applies them to each spatial position in the feature map. This enhances the network’s sensitivity to different channels and further improves the representation capability of features, as shown in Figure 4.

Neural Architecture Search (NAS) methods utilize an automated search process to discover the optimal network structure and parameter configuration. By employing reinforcement learning algorithms and search strategies, MobileNetV3 explores different combinations of network structures, including the number of layers, width diversity, and branching structures, among others, to optimize performance. The automated search process of NAS alleviates the burden of manual network design, enhances search efficiency, and improves performance. This enables MobileNetV3 to automatically search for the best network structure and parameter configuration, leading to better performance and results.

### 3.2. Combining with the Adaptive Neck of SA-Net

SA-NET [26] (Shuffle Attention for Deep Convolutional Neural Networks) is a deep convolutional neural network based on attention mechanisms. It aims to improve the network’s representational power and feature selection ability to better capture important features in images. The core idea of SA-NET includes grouped convolution and channel shuffling to enhance interactions between features, and it utilizes attention modules to adaptively adjust the weights of feature maps.

In traditional deep convolutional neural networks, convolution operations are usually performed simultaneously across all channels, resulting in independence between different channels in the feature maps. To enhance interactions between features, SA-NET introduces the concept of grouped convolution. It divides the input feature maps into multiple groups and performs independent convolution operations on each group. Specifically, when using grouped convolution, the input feature maps are divided into g groups, each containing c/g channels. Assuming the input feature map is *X*, the parameters of grouped convolution are denoted as *W*, and the output feature map is *Y*. The calculation formula for grouped convolution can be represented as follows:

(6) $$Y=Concatenate\left(\left[Conv\left(X_{1},\;W_{1}\right),\;Conv\left(X_{2},\;W_{2}\right),\;\ldots ,\;Conv\left(X_{G},\;W_{G}\right)\right]\right)$$

In this context, Conv represents the standard convolution operation, $X_{i}$ represents the input feature map i of the group, and $W_{i}$ represents the convolutional kernel associated with the group i. The purpose of this approach is to gradually capture specific semantic responses in each sub-feature map during the training process. Subsequently, individual convolution operations are performed on each subset, and their outputs are concatenated to form the final output feature map, enabling better fusion and transmission of feature information among different groups. Additionally, grouped convolution offers advantages in significantly reducing the computational complexity and parameter count of the model. Compared to traditional convolution operations, grouped convolution decomposes the convolution operation into smaller operations, with each operation handling only a subset of the channels. This approach reduces the computational load of each convolution operation and fully leverages the capabilities of parallel computing. The grouped processing of convolution operations also decreases the level of parameter sharing, thereby reducing the parameter count of the model.

Furthermore, SA-NET introduces channel shuffling to enhance the interaction between features. Channel shuffling rearranges the results of grouped convolution, allowing feature maps from different groups to interleave with each other. Through channel shuffling, information between different channels can interact and fuse more effectively. Adjacent channels originate from different groups, enriching the correlation between features and aiding in capturing more feature information and patterns. This operation increases the expressive power of the model while reducing computational and parameter requirements to a certain extent, resulting in a more lightweight network. Assuming the input feature map is $X_{i}$, and the output feature map after grouped convolution is Y, the channel shuffling operation can be represented as follows:

(7) $$Y=Concatenate\left(\left[Shuffle\left(X_{1}\right),\;Shuffle\left(X_{2}\right),\;\ldots ,\;Shuffle\left(X_{G}\right)\right]\right)$$

In this context, shuffle represents the channel shuffling operation; $X_{i}$ represents the input feature map i of the group. Channel shuffling has been widely applied in lightweight network architectures, particularly in mobile devices and embedded systems, to provide high-performance computation and recognition capabilities. It is an effective design strategy that reduces computational and storage requirements while maintaining model accuracy, making it suitable for various computer vision tasks.

Channel attention aims to capture the dependencies between different channels to better control the representational capacity of feature maps. In the SA network, for each sub-feature map, the global average pooling (GAP) operation is applied to compute the average along the spatial dimensions, obtaining the global statistics of the channels:

(8) $$s=\frac{1}{H\times W}\sum_{i=1}^{H}\sum_{j=1}^{W}Xk1(i,j)$$

Next, a simple gating mechanism is used to generate channel weights. Specifically, the parameters $W_{1}$ and $b_{1}$ are used for linear transformation, followed by a sigmoid activation function to obtain the weight parameter:

(9) $$weights=\sigma (W_{1}\cdot s+b1)$$

Finally, the weight parameter is applied to Xk1 linearly transforming the sub-feature map, resulting in the final output $X_{0}k1$ of channel attention. This step can be represented as:

(10) $$X_{0}k1=weights\cdot Xk1$$

Through this computation process, channel attention can model the importance of different channels and adjust the representation of sub-feature maps based on the weight parameters. This helps to enhance the model’s representational capacity and better capture the correlations between features.

**Figure 5:**
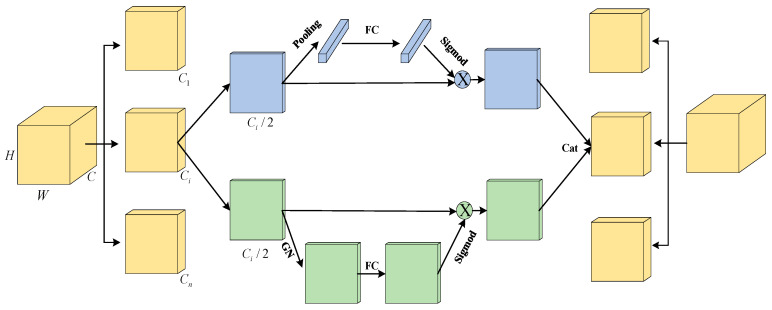
SA-net.

Spatial attention is used to determine which positions in the feature map contain informative content. In the SA network, the computation process of spatial attention is as follows: firstly, for each sub-feature map Xk2, its spatial statistics are obtained by applying group normalization (GN) operation, which enhances the representation capability of the feature map.

(11) $$GN(Xk2)=\frac{\left(Xk2-\mu \right)}{\sqrt{\sigma^{2}-\varepsilon }}$$

where Xk2 represents the sub-feature map, μ represents the mean within each group Xk2, σ represents the standard deviation within each group Xk2, and is a small constant for numerical stability. The adjusted feature map  ^Xk2 is further enhanced by applying function Fc(·):

(12)  ^Xk2=Fc(GN(Xk2))

where Fc(·) is a non-linear transformation function, such as the ReLU activation function. The aggregation of the adjusted sub-feature maps X0k1 and X0k2 is achieved by concatenating them:

(13) $$X0k=\left[X0k1;\;X0k2\right]$$

where X0k1 is the sub-feature map adjusted by channel attention, and X0k2 is the sub-feature map adjusted by spatial attention. Finally, the output of the SA module has the same size as the original feature map, which allows easy integration of the SA module with modern architectures, as shown in Figure 5.

### 3.3. C3_DS_Conv

Deep neural networks have achieved tremendous success in computer vision, natural language processing, and other fields. However, their complexity and large number of parameters lead to high computational and storage requirements. The main goal of quantization techniques is to reduce the precision of parameters and activation values, thereby reducing the computational and storage demands. Traditional deep neural networks use 32-bit floating-point numbers to represent parameters and activation values, while quantization techniques can represent them as lower-precision integers or binary forms. For example, parameters and activation values can be represented using 8-bit integers or binary, significantly reducing the computational and storage overhead. Quantization serves multiple purposes. Firstly, it can reduce computational costs. Lower-precision representations can decrease the complexity of multiplication and addition operations, speeding up inference, which is crucial for real-time applications and resource-constrained devices such as mobile devices and embedded systems. Secondly, quantization can reduce storage costs. Deep neural networks typically have a large number of parameters, requiring significant memory space for storage. Through quantization, parameters can be represented in compact integer or binary form, significantly reducing storage requirements, which is important for deploying and running deep neural networks in resource-constrained environments. Additionally, quantization can improve energy efficiency. By reducing computational demands, quantization can reduce energy consumption, prolong battery life, and enhance device efficiency, which is beneficial for power-sensitive applications like mobile devices and wireless sensor networks.

During the inference process of neural networks, adopting quantization methods with low-precision representations can significantly reduce computational and storage overhead. However, how to quantize weights and activations without sacrificing accuracy remains a challenge. DS_Conv [30] employs a strategy called block-wise quantization, where weights and activations are divided into different blocks and quantized separately. By converting floating-point values into fixed-bit integer values and using floating-point scaling factors to preserve the quantized precision, it achieves low-precision representation without compromising the accuracy of the network. The core principle of it is based on the relative distribution invariance of quantized weights and activations. It utilizes the block-wise strategy in quantization operations, representing weights and activations using integer values and employing floating-point scaling factors to maintain the quantized precision. By minimizing the Kullback–Leibler (KL) divergence or L2 norm, it computes the scaling factor for each block to re-map the quantized values back to the original range.

**Figure 6:**
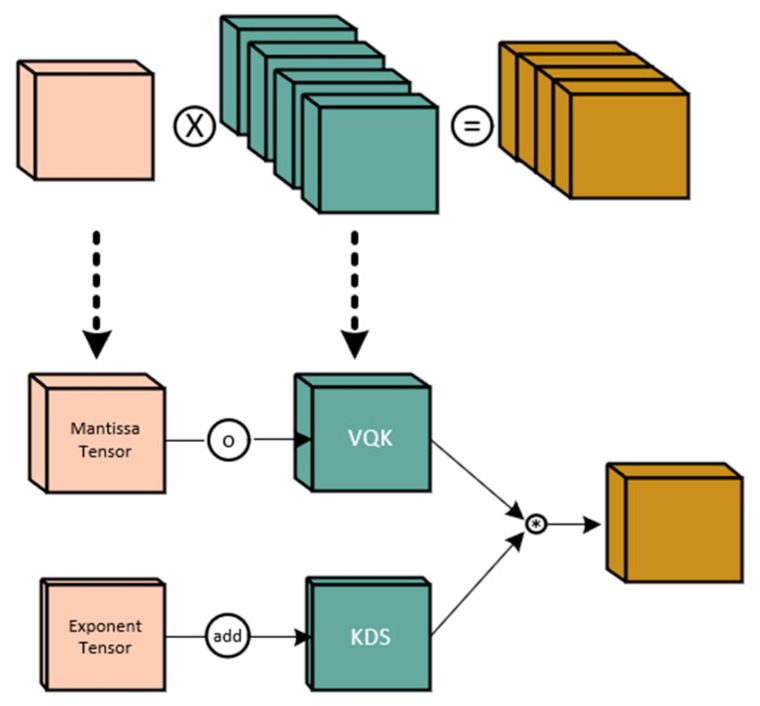
DS_Conv.

The specific structure of it consists of two key components: Variable Quantization Kernel (VQK) and Kernel Distribution Shifting (KDS), as shown in Figure 6. VQK is an integer tensor of the same size as the original weight tensor, represented in 2’s complement format, with its range determined by a preselected number of bits. KDS is a floating-point tensor used to store the scaling factors for each block. By multiplying the integer values of weights and activations with their respective scaling factors, the distribution of each block is readjusted to the correct range. It also employs an activation quantization method called Block Floating-Point (BFP). It partitions the activation tensor into blocks and performs clipping and shifting on other activations based on the maximum exponent within each block. This allows the use of fewer bits in the activation tensor and enables low-precision integer operations between weights and activations.

By appropriately selecting the values of block size B and number of bits b, it achieves a trade-off between computational and storage efficiency and inference accuracy. A larger block size B can reduce storage overhead and the depth of KDS but may increase clipping errors. A smaller number of bits b can lower storage costs but may result in greater computational complexity.

It is a quantization method for neural network inference that achieves low-precision representation without sacrificing network accuracy through block-wise quantization and floating-point scaling factors. It employs the two key components, VQK and KDS, to store quantized weights and activations, and utilizes the BFP method for activation quantization. By appropriately selecting the block size and number of bits, it strikes a balance between computational and storage efficiency and inference accuracy. This approach demonstrates good performance even without training data and has broad prospects for applications.

## 4. Experimental Results and Analysis

### 4.1. Data Collection and Processing

**Figure 7:**
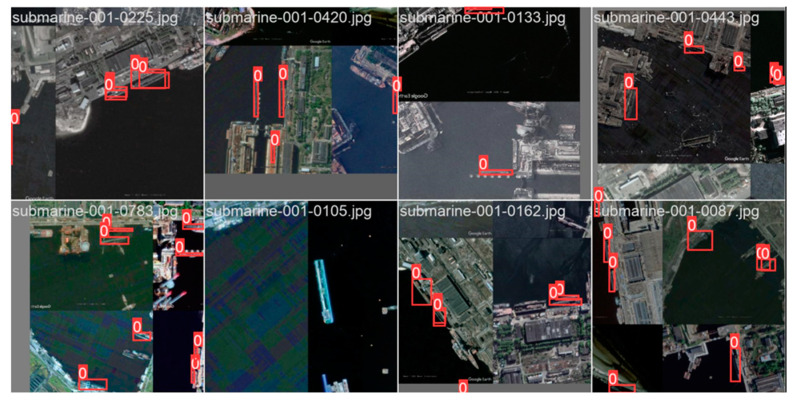
Data enhancement display.

The dataset used in this experiment consists of full-color images collected from a port, with a resolution of 1024 × 1024 × 24. The dataset comprises a total of 1000 images, and the labels are in Pascal VOC format (XML). The values for hsv_h, hsv_s, and hsv_v are set to 0.015, 0.7, and 0.4, respectively. The horizontal or vertical translation ratio of the images is set to 0.5, and there is a 0.5 probability of horizontally flipping the images. The mosaic data augmentation technique is employed, as shown in Figure 7. The purpose of applying image augmentation methods is to increase the diversity of data during the training phase, thereby improving the performance and robustness of the object detection model. By applying various transformations and perturbations to the images, it is possible to simulate various situations and scenarios in the real world, enabling the model to better adapt to different environments and variations.

### 4.2. Training Parameter Setting

In order to achieve a fair experimental comparison, no pre-trained weight files were loaded during both the original training phase of YOLOv5 and the improved network structure. The YOLOv5s model was used as the baseline for the experiments. The number of epochs was set to 80, with a batch size of 32. The learning rate was dynamically adjusted using cosine annealing, with an initial learning rate of 0.0036 and a momentum parameter of 0.937. The weights for bounding box loss, object loss, and binary cross-entropy loss for object classification were set to 0.5, 1.0, and 0.1, respectively. The loss function includes bounding box loss, class loss, object presence loss, and object absence loss.

**Figure 8:**
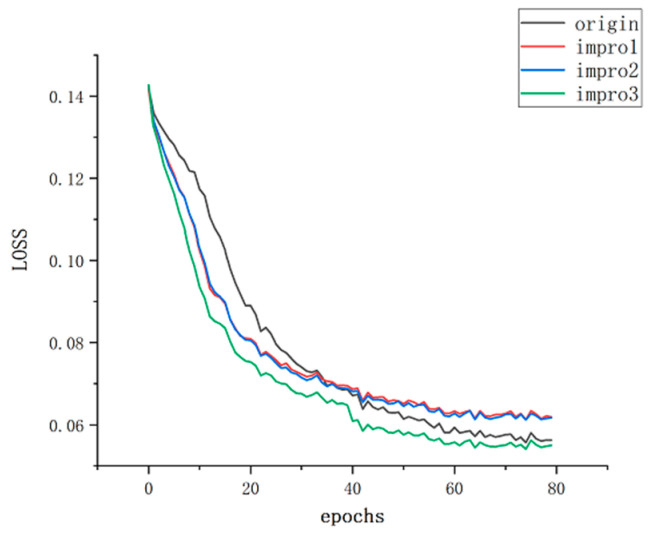
LOSS before and after improvement.

The bounding box loss is measured using the Smooth L1 loss, which evaluates the model’s accurate prediction of bounding box positions. The class loss uses the cross-entropy loss function to measure the model’s accurate prediction of target classes. The object presence loss encourages the model to make accurate predictions regarding the presence of objects using binary cross-entropy loss, while the object absence loss utilizes the negative log-likelihood loss function to reduce false positives. By simultaneously optimizing these loss functions, the YOLOv5 model can achieve accurate object detection, accurately predicting bounding box positions, target classes, and object presence. The weights of the loss functions can be adjusted based on the requirements of the task to balance the contributions of different losses, thereby improving the model’s performance and robustness. With the parameter settings and loss optimization mentioned above, the training of the model before and after improvement on the same dataset resulted in the relationship between loss and epochs as shown in Figure 8.

### 4.3. Results

The experimental results are an essential part of evaluating and comparing the performance of the YOLOv5 model before and after improvement in the object detection task. Next, we will thoroughly present the experimental results, including performance metrics of the models before and after improvement (such as accuracy, recall rate, average precision, lightweightness, etc.), and conduct comparisons and analysis.

#### 4.3.1. Overall Performance

**Figure 9:**
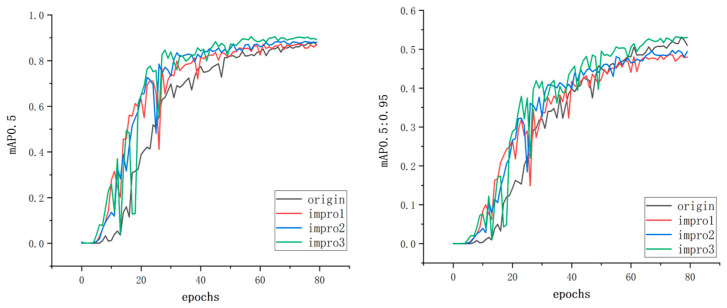
mAP0.5 and mAP0.5:0.95.

By plotting the curves of mAP0.5 and mAP0.5:0.95 metrics, the impact of the improvement on the model’s performance can be clearly demonstrated, as shown in Figure 9.

First, let us focus on the mAP0.5 metric, which measures the accuracy of the model at a relatively relaxed prediction threshold. By observing the curves before and after the improvement, we can see a significant improvement in the performance of the model in terms of mAP0.5. This means that the improved model has higher accuracy in detecting objects and can better locate and recognize object bounding boxes. On the other hand, the mAP0.5:0.95 metric measures the accuracy of the model at a stricter prediction threshold, requiring more precise detection of objects. By observing the curves before and after the improvement, we can also observe a significant improvement in the performance of the improved model in terms of mAP0.5:0.95. This means that the improved model can maintain high precision at a higher recall rate and predict the position and category of objects more accurately.

Overall, by comparing the curves of the model’s performance before and after the improvement in terms of mAP0.5 and mAP0.5:0.95, we can clearly observe the positive impact of the improvement on the model’s performance. These results validate the effectiveness of the improvement method and demonstrate its significant effect in enhancing object detection performance in the YOLOv5 model. These findings further support and strengthen our arguments regarding the reliability and effectiveness of the improvement method.

#### 4.3.2. Better Performance

Here is a specific numerical comparison of the improvement effect on four models before and after improvement in terms of P (Precision), R (Recall), and mAP0.5 (Mean Average Precision):

As shown in the Table 1, the models show significant improvements in terms of P, R, and mAP0.5 after the improvement. P reflects the accuracy of the model’s predictions, indicating how many of the predicted targets are actually true targets. Through the improvement, the P values of the models have increased to varying degrees, indicating that the improved models can predict targets more accurately and reduce false positives. In terms of R, the models before and after the improvement also show significant improvements. R measures the model’s ability to detect true targets, indicating how many true targets the model can correctly identify. Through the improvement, the R values of the models have significantly increased, indicating that the improved models can detect targets more comprehensively and improve recall. mAP0.5 measures the overall detection accuracy of the model at a relatively relaxed prediction threshold. Through the improvement, the mAP0.5 values of each model have significantly increased, indicating that the improved models have higher accuracy and localization ability in detecting targets. In conclusion, by comparing the specific numerical improvement effects of the four models before and after the improvement in terms of P, R, and mAP0.5, we can clearly observe the positive impact of the improvement on the model’s performance. These results further validate the effectiveness of the improvement method and demonstrate that the introduced improvements in the YOLOv5 model significantly enhance the accuracy, recall, and overall performance of object detection.

#### 4.3.3. More Lightweight

In addition to the improvements in performance metrics, the improved model also demonstrates outstanding results in terms of lightweightness. Here is a comparison of the models before and after improvement in terms of network layers, parameters, and computational complexity:

As shown in Table 2, Firstly, in terms of parameter count, the improved model significantly reduces the number of parameters required. Reducing the parameter count helps decrease the storage space requirements and computational complexity of the model, making it more lightweight. Despite the reduction in parameter count, the improved model is still able to maintain good detection performance, further validating the effectiveness of the lightweight improvement.

Secondly, in terms of computational complexity, the improved model also significantly reduces the computational requirements. By reducing the parameter count, the improved model can reduce the computational resource demands while maintaining high performance. This is particularly important for deploying the model on resource-constrained devices such as embedded systems or mobile devices.

Although the improved model has an increase in the number of network layers, it still demonstrates remarkable lightweight performance. The increase in network layers may increase computational and storage requirements, but the improved model, through optimized design and parameter reduction, can still provide excellent object detection performance under lightweight conditions.

In conclusion, the improved model has shown outstanding results in terms of lightweight design. By reducing the parameter count and computational complexity, the improved model can significantly reduce the complexity and resource requirements while maintaining high performance. This makes the improved model have broader application prospects in lightweight scenarios and provides an efficient object detection solution for resource-constrained devices.

#### 4.3.4. Focus More on the Outlook

**Figure 10:**
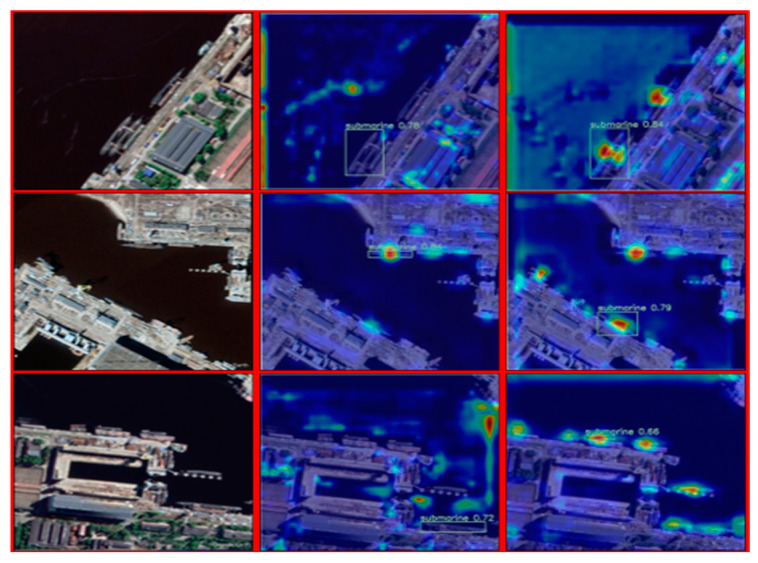
Model test effect before and after improvement.

The adaptive mechanism allows the network to dynamically adjust the weights and representations of features based on the characteristics of the input data. Its purpose is to enhance the model’s focus on important target regions and reduce attention to background or minor regions, thereby improving the accuracy and robustness of object detection. Specifically, we used a heatmap-based approach to visualize the performance of the network before and after improvement, as shown in Figure 10. The heatmap provides an intuitive display of the model’s attention to different regions, with brighter areas indicating higher model focus on those regions.

By comparing the heatmaps before and after improvement, we can observe that the improved model exhibits higher attention to the target regions. This means that the improved model can more accurately locate and recognize objects, making it more robust in complex backgrounds or occlusion situations. It helps the model focus on critical targets and improves detection precision and recall. At the same time, by reducing attention to the background and minor regions, it also reduces false detections, thereby enhancing the model’s robustness and reliability.

In conclusion, by employing an adaptive improvement scheme for the network and visualizing the network’s performance before and after improvement using heatmaps, we observed that the improved model exhibits stronger attention to target regions. This significantly enhances the accuracy and robustness of object detection tasks.

#### 4.3.5. Excellent Performance in Practice

This section will focus on showcasing the outstanding performance and improvement of the model in the object detection task after the improvement. We present the accuracy, confidence, and improvement regarding the issue of false negatives by comparing the well-annotated dataset with the dataset predicted by the original model, as well as the predicted results of the improved model.

**Figure 11:**
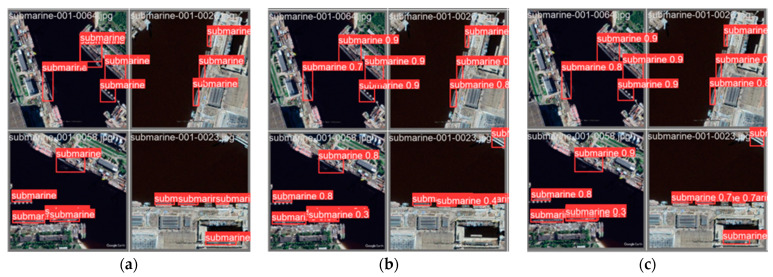
Display the results before and after improvement. (**a**) Original label file, (**b**) original Yolov5 model, and (**c**) test results of improved YOLOv5.

By comparing the well-annotated dataset with the dataset predicted by the original model and the predicted results of the improved model, as shown in Figure 11, we can visually observe the superior performance of the improved model in the object detection task. Firstly, the improved model can accurately detect objects in the well-annotated dataset and provide predictions with higher confidence. Compared to the original model, the improved model can more accurately localize objects and classify them correctly, indicating higher accuracy and confidence. Secondly, we can observe that the issue of false negatives that may exist in the original model has been effectively addressed or reduced in the improved model. The improved model can better recognize and capture objects, ensuring successful prediction and annotation of previously missed objects. This improvement can be visually observed in the predicted results of the improved model. Compared to the original model, the improved model can detect objects more comprehensively, reducing the occurrence of false negatives and providing more complete and accurate object detection results.

#### 4.3.6. Typical Algorithm Analogy

In order to highlight the effectiveness of the improvements made to YOLOv5 in this study, we conducted comparisons with other object detection algorithms, including Faster R-CNN, Single Shot Multibox Detector (SSD), YOLOv3, and the enhanced algorithm proposed in this paper.

Faster R-CNN is an object detection algorithm based on region proposal networks, with higher detection accuracy but relatively slower processing speed. We compared the enhanced algorithm in this study with Faster R-CNN to validate the advantages of the improved algorithm in terms of both accuracy and speed.

SSD is a one-stage object detection algorithm known for its faster detection speed, but it may have limitations in detecting small objects. We compared the enhanced algorithm in this study with SSD to showcase its performance in detecting small objects. YOLOv3 is an earlier version of the YOLO series, and it exhibits certain differences in speed and accuracy compared to YOLOv5. We compared the enhanced algorithm in this study with YOLOv3 to emphasize the improvements achieved over the earlier version. This is shown in Table 3, we compared the enhanced algorithm in this study with YOLOv5 to showcase its performance in terms of both accuracy and speed.

Through these comparative experiments, we aim to establish the evident advantages of the improved algorithm over other algorithms in object detection tasks, thereby highlighting the effectiveness of the proposed enhancements.

## 5. Conclusions

This paper achieved significant performance improvement in submarine target detection through the improvement of the object detection model. Compared to the original model, the improved model showed an 8.54% increase in precision (P), a 6.02% increase in recall (R), and a 3.36% increase in mean average precision (mAP0.5). This indicates a significant improvement in accuracy in the improved model for object detection. Additionally, this paper emphasizes the importance of lightweight design in the model. The improved model reduced the parameter count by 34.1% and the computational complexity by 67.9% compared to the original model, achieving a notable lightweight effect. This enables the improved model to perform efficient object detection in resource-constrained environments. Furthermore, this paper introduces an attention mechanism to enhance the interaction between features and visually demonstrates the improvement of the improved model in terms of confidence and the issue of missed detections. By adaptively adjusting the weights of the feature maps, the improved model can accurately focus on important features, thereby improving the accuracy and robustness of object detection.

In summary, this paper has achieved a dual improvement in accuracy and lightweight design in the task of submarine target detection through the improvement of the model’s design and performance analysis. The improved model not only significantly improves accuracy but also enables efficient object detection in resource-constrained environments through lightweight design. These research findings are significant for enhancing the accuracy and efficiency of object detection and provide valuable references for the development of future lightweight object detection algorithms.

## Figures and Tables

**Table 1:** Comparison of evaluation indexes of the model before and after improvement. An upward numeric arrow indicates that the value has improved from the original value.

Model	P	R	mAP0.5
origin	0.822	0.769	0.874
impro1	0.807	0.79	0.873
impro2	0.874	0.75	0.882
impro3	0.893↑	0.815↑	0.903↑

**Table 2:** Comparison of lightweight degree of the model before and after improvement. An upward numeric arrow indicates that the value has improved from the original value. A downward digital arrow indicates that the value is reduced from the original value.

Model	Layers	Parameters	GFLOPS
origin	214	7,022,326	15.9
impro1	294	4,694,678	7.0
impro2	320	4,599,702	5.0
impro3	298↑	4,627,590↓	5.1↓

**Table 3:** Typical Algorithm Analogy.

Model	P	R	mAP0.5/%
Faster-RCNN	86.1	79.2	68.7
SDD	84.9	75.3	72.8
YOLOv3	80.4	81.2	84.1
YOLOv5	82.2	76.9	87.4
Ours	89.3	81.5	90.3

## Data Availability

The dataset used in this study is not available for public access due to confidentiality or other restrictive reasons.
